# Diagnostic role of magnetic resonance angiography in Swyer James syndrome: Case series of two cases

**DOI:** 10.4103/0970-2113.68326

**Published:** 2010

**Authors:** Umesh C. Parashari, Ragini Singh, Anit Parihar, Pallavi Aga, Rajesh Yadav

**Affiliations:** *Department of Radiodiagnosis, CSM Medical University (Upgraded K.G. Medical University), Lucknow, India*

**Keywords:** Bronchiolitis obliterans, MR angiography, pruned tree appearance, unilateral hyperlucent lung

## Abstract

Swyer James syndrome is a rare syndrome which occurs due to viral illness in early childhood. The post infective obliterative bronchiolitis results in arrest of lung growth and alveolarization with reduced vascularity resulting in classical radiological features. We describe two cases of patients fulfilling all the criteria of the syndrome - 1) Unilateral hyperlucent small lung in chest radiograph with air trapping on expiration, small ipsilateral hila and pulmonary artery. 2) Diffuse decrease in attenuation of lung parenchyma with bronchiectasis and reduction in vascularity. 3) Unilateral pruned tree appearance on angiography (MRA). The clinical presentation was recurrent chest infection in a child and infrequent bouts of hemoptysis in a middle aged female. The study demonstrates the role of magnetic resonance angiography in diagnosing the condition.

## INTRODUCTION

We report a case series of two cases of Swyer James syndrome.[1] The syndrome is rare and occurs following viral insult in infancy or early childhood. The infective insult leads to acute obliterative bronchiolitis resulting in arrest of progressive alveolarization and proper development of lung. The syndrome results in reduced vascularity with paucity of bronchial subdivisions (cut off at 4 ^th^ to 5 ^th^ generation). The study was performed to rule out the cause of recurrent respiratory infection in first patient and infrequent bouts of hemoptysis in another patient.

## CASE REPORTS

### Case 1

A 13-year-old female child presented with recurrent attacks of respiratory tract infection for last few years. Physical examination revealed presence of crepts in almost whole of left lung. Arterial blood gas analysis showed reduced oxygenation. The pulmonary function test showed diminution of flow with reduced FEV1.

**Figure 1:**
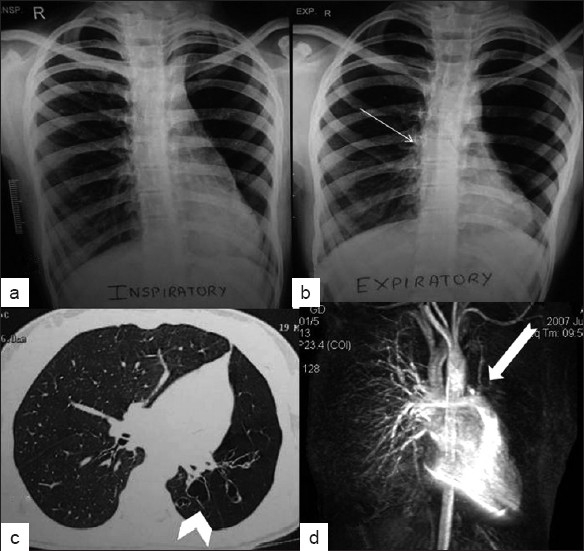
Inspiratory (a) and expiratory (b) chest radiograph of 13 year old girl showing small hyperluscent left hemithorax with mild mediastinal swing towards normal (right) side(thin white arrow). HRCT (c) demonstrates decrease in volume of left lung with decrease in bronchovascular markings on left side with bronchiectatic changes (white arrow head). MR angiography (d) confirming the diagnosis by revealing smaller pulmonary artery on left side with typical pruned tree appearance (thick white arrow)

The radiological evaluation started with inspiratory chest radiograph, which showed small hyperluscent left hemithorax with evidence of air trapping on expiratory radiograph. The left hilum was small. Slight hyperinflation of right lung was also noted [Figure 1a and b]. High resolution CT (HRCT) was done following chest radiograph, which showed small left hemithorax with diffuse decrease in attenuation. Paucity of broncho vascular markings with proximal bronchiectasis was noted in left hemithorax. The pulmonary artery was smaller on the left side[Figure 1c]. Magnetic resonance (MR) angiography was performed after HRCT revealed typical pruned tree appearance on left side confirming the diagnosis of Swyer James syndrome [Figure 1d].

### Case 2

**Figure 2:**
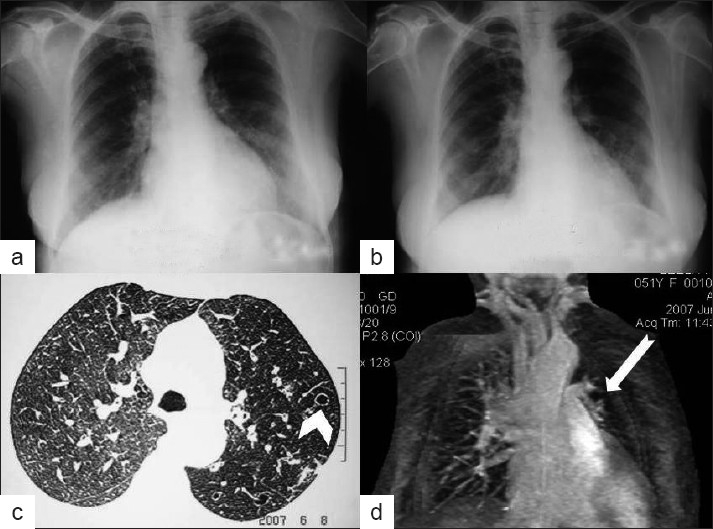
Inspiratory (a) and expiratory (b) chest radiographs of 51-year-old female showing small hyperluscent left hemithorax with air trapping on expiration. HRCT (c) shows reduced volume of left lung with decrease in parenchymal attenuation and bronchovascular markings on left side. Bronchiectatic areas are seen in left hemithorax (white arrow head). MR angiography (d) shows typical pruned tree appearance on left side (thick white arrow)

A 51-year-old woman presented with infrequent bouts of hemoptysis for approximately 10 years. Hemoptysis was streaky in nature. Patient did not have any other complaint. Physical examination revealed presence of crepts in some areas in left hemithorax. The arterial blood gas analysis showed slightly reduced oxygenation. On pulmonary function test, diminution of flow with reduction of FEV1 was noted. The PA view chest radiograph showed small hyperlucent left hemithorax with evidence of air trapping on expiration. Small left hilum with few bronchiectasis changes were also noted along with slight hyperinflation of right lung [Figure2a and b]. HRCT lung showed small left hemithorax with diffuse decrease in attenuation, proximal bronchiectasis with paucity of broncho vascular markings. The pulmonary artery was smaller on the left side [Figure 2c]. MR angiography showed typical pruned tree appearance on left side confirming the diagnosis [Figure 2d].

## DISCUSSION

The Swyer James syndrome is also called Macleod syndrome/ Bret’s syndrome / Janus syndrome in honor of workers who initially described this rare entity.[2,3] They also demonstrated its association with Fallot’s tetrology. The condition arises as a result of some viral insult in infancy or childhood. The agents which are implicated are adenovirus, respiratory syncital virus, influenza virus, Mycoplasma pneumoniae, *Streptococcus pneumoniae* and *Staphylococcus.* In the initial eight years of life the lung growth occurs by progressive alveolarization and later on the growth occurs by expansion of preexisting bronchi.

The infective insult during infancy or childhood results in post infectious acute obliterative bronchiolitis (usually developing after six months to three years), which causes arrest of growth and alveolarization leading to hypoplasia of affected lung with reduced vascularity with paucity of bronchial subdivisions (cut off at 4 ^th^ to 5 ^th^ generation). Proteases released by phagocytes may be causative for elastolysis. Increase in CD8+ cells is also noted in broncho alveolar lavage of patients suffering from Swyer James syndrome. The disease usually affects one lung; however, both lungs and part of the lung may be involved. The affected lung or portion of the lung will not develop properly and will be smaller than its counterpart along with evidence of air trapping, resulting in unilateral hyperlucency. Finally, fibrous obliteration of airway lumen occurs.

The usual presentation of the patient is recurrent bouts of cough, fever, dyspnoea, which may be exertional. Some times the patients may present with history of hemoptysis, as in one of our cases. Weight loss may also be present. The syndrome may be complicated by spontaneous multi vessel coronary dissection.[4] The Swyer James syndrome results in chronic lung illness with abnormal lung dynamics during inspiration and expiration (demonstrated as abnormal time attenuation curves during inspiration and expiration) with air trapping which increases on expiration with bronchial and bronchiolar abnormalities. Placental transmogrification of lung has been described recently in patients of Swyer James syndrome,[5] structures resembling placental villi in lung parenchyma were described.

After the initial infective insult radiographic findings appear after months to years. Usual investigations performed are chest radiograph, high resolution computed tomography (both of these investigation should be done in inspiration and expiration), MRI, angiography, ventilation perfusion scanning.

Chest X-ray is the initial modality of investigation which demonstrates small hyperlucent affected lung/ region with compensatory hyperinflation of contra lateral lung. Evidence of air trapping may be noted in expiration which is a *sine qua non* for making diagnosis of Swyer James syndrome. Swing of mediastinal may be noted towards the normal lung on expiration. The hila on the affected side will be smaller. Evidence of bronchiectasis, scarring, and irregular pulmonary vasculature may be noted. Excursions of hemi diaphragm will also be markedly asymmetrical. Brochography may show dilated bronchi with sharply terminated segments. It is rarely used now a days.

HRCT is done with thin collimation in both phases of respiration to demonstrate air trapping. For proper demonstration of mosaic pattern prone position may be required. There will be evidence of diffuse decrease in attenuation of lung parenchyma on the affected side which will be smaller in size with reduction in the broncho vascular markings and smaller ipsilateral pulmonary artery. Paucity of bronchial subdivisions, proximal bronchiectasis and expiratory air trapping are best appreciated on HRCT.[6]

MRI itself does not contribute to the final diagnosis; however, MR angiography demonstrates smaller pulmonary artery and its branches on the affected side. The narrowed attenuated arteries coursing through the radiolucent lung will produce “pruned tree appearance”.

Matched ventilation perfusion defects may be demonstrated by ventilation perfusion scanning due to abnormal development of pulmonary vasculature and lung parenchyma. Marked air trapping may also be noted in the washout phase.[7] Scintigraphy may demonstrate findings of Swyer James syndrome in absence of significant radiological signs with demonstration of additional areas of involvement which will be normal on radiograph.

The important differential diagnosis of the Swyer James syndrome are congenital lobar emphysema, congenital hypoplasia of the lung, hypoplastic pulmonary artery, compensatory unilateral emphysema secondary to lobectomy, pulmonary embolic disease, pneumothorax, foreign body in air way.[8]
